# Challenges and Opportunities in the Oral Delivery of Recombinant Biologics

**DOI:** 10.3390/pharmaceutics15051415

**Published:** 2023-05-05

**Authors:** Solene Masloh, Maxime Culot, Fabien Gosselet, Anne Chevrel, Leonardo Scapozza, Magali Zeisser Labouebe

**Affiliations:** 1 Laboratoire de la Barrière Hémato-Encéphalique (LBHE), Faculté des sciences Jean Perrin, University of Artois, UR 2465, Rue Jean Souvraz, 62300 Lens, France; solene.masloh@unige.ch (S.M.); fabien.gosselet@univ-artois.fr (F.G.); 2 Affilogic, 24 Rue de la Rainière, 44300 Nantes, France; anne@affilogic.com; 3 School of Pharmaceutical Sciences, University of Geneva, 1 Rue Michel Servet, 1201 Geneva, Switzerland; leonardo.scapozza@unige.ch; 4 Institute of Pharmaceutical Sciences of Western Switzerland, University of Geneva, 1 Rue Michel Servet, 1201 Geneva, Switzerland

**Keywords:** biologics, oral delivery, intestinal mucosa, permeability, stability, in vitro, ex vivo

## Abstract

Recombinant biological molecules are at the cutting-edge of biomedical research thanks to the significant progress made in biotechnology and a better understanding of subcellular processes implicated in several diseases. Given their ability to induce a potent response, these molecules are becoming the drugs of choice for multiple pathologies. However, unlike conventional drugs which are mostly ingested, the majority of biologics are currently administered parenterally. Therefore, to improve their limited bioavailability when delivered orally, the scientific community has devoted tremendous efforts to develop accurate cell- and tissue-based models that allow for the determination of their capacity to cross the intestinal mucosa. Furthermore, several promising approaches have been imagined to enhance the intestinal permeability and stability of recombinant biological molecules. This review summarizes the main physiological barriers to the oral delivery of biologics. Several preclinical in vitro and ex vivo models currently used to assess permeability are also presented. Finally, the multiple strategies explored to address the challenges of administering biotherapeutics orally are described.

## 1. Introduction

Recombinant biological molecules represent a new category of drugs commonly referred to as biologics or biotherapeutics and defined as large and complex molecules. Contrary to conventional small drugs synthetized through chemical reactions, they are produced using genetic engineering techniques and are typically derived from living organisms [1]. The development of these therapeutic molecules, including peptides and proteins, has rapidly gained momentum in the past decade and they now represent the fastest growing sector in the pharmaceutical industry. Indeed, since 2014, and out of a total of 364 drugs [2], 96 biologics were accepted by the center for drug evaluation and research with an all-time record number of 17 novel biotherapeutics approved in 2018 [3]. Their advantages, such as their high selectivity, their potent responses, and their limited side effects, have revolutionized multiple disease treatments. Their use has delayed or reversed the course of immune-related illnesses, changed the lives of people with rare diseases, and has offered hope to many patients who previously had no effective treatment options for their condition.

Despite the demonstrated benefits of biologics, the majority are formulated for parental delivery. However, although injections allow the achievement of a high bioavailability, they are also associated with multiple drawbacks leading to poor patient compliance such as pain, reactions at the injection site, discomfort, or the need of medical assistance [4,5]. These limitations make the non-invasive and pain-free oral administration the most convenient and preferred method for drug delivery [6].

Gastro-intestinal (GI) barriers, including the intestinal epithelium restricting the passive transport of large molecules [7,8] and the harsh environment of the tract, prevent biological drugs to attain an effective therapeutic concentration in blood. In-depth understanding of the parameters influencing bioavailability is a key to improve the design and development of orally delivered biotherapeutics [9]. Hence, in vivo experiments became an inevitable step to investigate the mechanisms of transport and to ensure that a molecule is safe and effective in a living biological system. However, ethical considerations and animal protection [10] encouraged the development of alternative models mimicking the human physiology. In this context, preclinical in vitro and ex vivo methodologies have been developed and optimized by the scientific community over the years to study drug permeability [11]. In vitro systems display several advantages: they are cheap, fast, and can be adapted to high throughput screening [12]. However, numerous examples of these models are lacking some crucial aspects of intestinal physiology. That is why, more complex ex vivo approaches using viable and functional extracted tissues or organs are employed in combination with these cell-based systems [13,14].

In parallel to the development of these models, different strategies have been investigated in order to improve the intestinal permeability and stability of biologics following an oral delivery. Some approaches focus on targeting a receptor to promote transcytosis [15,16,17], while others aim at increasing mucus interaction [18,19,20,21], improving absorption [22,23,24,25], enhancing stability [26,27], or other innovative systems. Several of these techniques are already used in marketed biological products destined to be ingested [23,28,29].

In this review, the main properties of the GI tract that need to be considered for the oral administration of biologics are described. Thereafter, this article provides an overview of some in vitro and ex vivo models that can be used to predict the human intestinal permeability of recombinant biological molecules. Finally, some of the strategies imagined to enable or improve the oral delivery of biologics are presented.

## 2. Challenges Associated with Oral Delivery of Drugs

**Figure 1:**
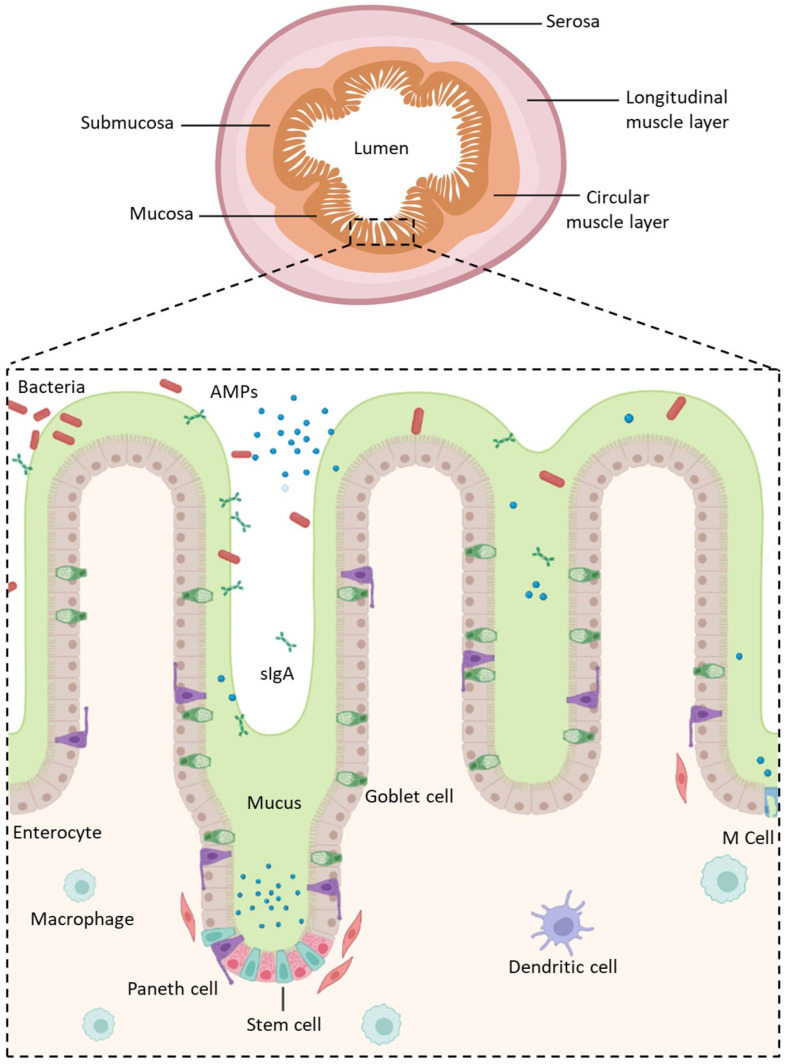
Structure of the intestinal epithelium. AMPs: Antimicrobial Peptides; sIgA: Secretory Immunoglobulin A. Created with Biorender.com (accessed on 24 January 2023).

The GI tract, a dynamic barrier of approximately 300 m^2^ [30], is divided into four concentric layers connected by connective tissues as well as neural and vascular networks: the mucosa, the submucosa, the muscularis, and the serosa (Figure 1) [31]. The intestinal mucosa covering the lamina propria with blood and lymphatic vessels is formed from a highly absorptive epithelium composed of several polarized cell types displaying distinct apical (lumen) and basolateral (serosal) membranes [11]. Its role is not only to be permeable in order to absorb nutrients, water, and electrolytes, and allow immune sensing, but also to act as an effective barrier by filtering out undesired and harmful molecules to prevent their transport into the bloodstream [32]. This semi-permeable barrier, the mucus, and the harsh conditions of the tract, represent the main obstacles to overcome in order to allow the oral delivery of biologics.

### 2.1. Gastro-Intestinal Barriers

#### 2.1.1. Mucus and Glycocalyx

When a molecule arrives into the gut lumen, the mucus, whose thickness varies depending on the region of the GI tract, represents the first physical barrier encountered [33] (thickest layers in the gastric and colonic segments [34]). This layer, which protects the mucosal surface of the GI tract and lubricates luminal content to ease the passage of food, is continuously secreted by tall columnar goblet cells and shed into the lumen. It acts as a semi-permeable barrier that captures digestive enzymes, acids, or toxins, to prevent their contact with the underlying epithelial cells, while allowing the uptake of nutrients, water, and other small compounds. The mucus is mostly composed of water, lipids, and mucins. These latter are glycosylated proteins that create extensive intermolecular interactions decreasing the movement of entrapped molecules by giving the mucus its viscoelastic nature [30,35,36]. Due to the constant turnover of mucus, the residence time of biologics is reduced into the small intestine [37].

Underneath the mucus, the glycocalyx, a carbohydrate highly charged cell coat, also represents a physical barrier to molecules trying to reach cell membranes. This filamentous network, atop intestinal epithelial cells (IECs), is an uniform layer covering enterocytes of the small and large intestines with a thickness varying from 60 nm to a micrometer [38]. It is composed of glycolipids and diverse glycoproteins whose majority are transmembrane mucins [39]. Mucus and glycocalyx compositions and functions lead to a stable system preventing the GI tract from being harmed by its own challenging conditions [40,41].

#### 2.1.2. The Intestinal Epithelium

The mucosa, the innermost tunic of the gut wall, has a crucial role in the transport of molecules and GI immunity. It consists of IECs connected by intercellular junctions that are located underneath the mucus/glycocalyx layers and resting over the underlying lamina propria. In the small intestine, the IECs form a highly absorptive epithelium organized into crypts and villi in order to increase the mucosal surface area and the absorption of nutrients. It is renewed every few days by the replication of undifferentiated pluripotent stem cells. However, villi are absent from the colon.

Pluripotent stem cells maturate in an heterogenous cell population with cell types having distinct and specific functions (Figure 1) [42,43] such as:

- Enterocytes, which are the predominant cell type found in the intestinal epithelium (90%). They have a microvilli network that increases their surface area for transport and forms a brush border on their apical surface. Many digestive enzymes, receptors, and transporters needed for the uptake and transport of molecules can be found on their microvilli [44];
- Goblet cells, which represent 10% of all IECs and are responsible for mucus production;
- Enteroendocrine cells, which are able to secrete and release intestinal hormones or peptides into the circulation upon stimulation [45];
- Long-lived Paneth cells, which produce and release antimicrobial peptides in the intestinal crypts of the monolayer [46];
- Microfold cells (M cells), which cover the luminal side of the lymphoid follicles of gut-associated lymphoid tissue [47] and are involved in the active transport of luminal antigens to the underlying lymphoid follicles in order to initiate an immune response [33].

The majority of the cell types located in the colon are also found in the small intestine, except Paneth cells and M cells which are found overlaying Peyer’s patches and are both unique to the small intestine [48].

**Figure 2:**
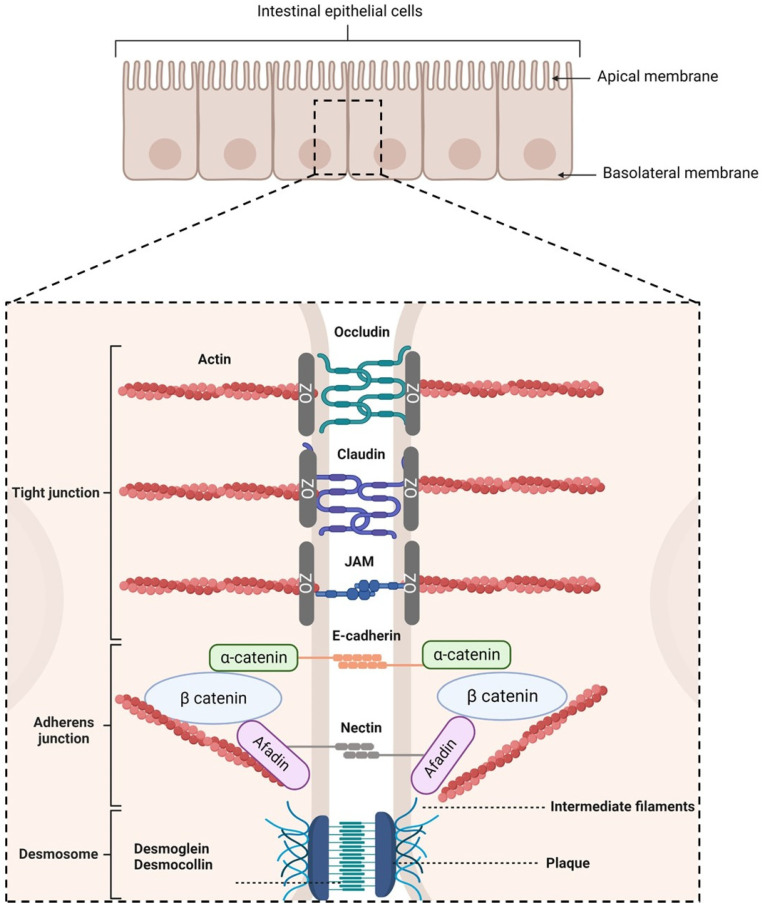
Schematic representation of intercellular junctions. Created with Biorender.com (accessed on 25 January 2023).

Following the ingestion of a molecule, two routes can lead to its transport into the systemic circulation depending on its shape, size, and charge [49]: the transcellular and the paracellular pathways. This latter is sealed by protein-adhesive contacts modulated by the intercellular junctions connecting the IECs. These junctions regulate intestinal permeability by inducing the absorption of essential molecules [50,51], while preventing the transport of harmful entities into systemic circulation [52]. They are separated into three regions, from the apical to the basolateral side of the cells (Figure 2):

1. Tight junctions (TJs), which are located at the most apical region of polarized IECs. They make paracellular transport mainly dependent on the size of a molecule due to their multiple protein–protein interactions between adjacent cells [53,54,55]. TJs are composed of transmembrane proteins (occludins, claudins, tricellulin, and junctional adhesion molecules) and plaque proteins (e.g., zonula occludens-1, -2, and -3), which act as bridges to connect integral membrane proteins to the actin cytoskeleton and to other signaling proteins. They are also composed of other regulatory proteins [56].
2. Adherens junctions (AJs), which are located beneath TJs. They are involved in cell–cell adhesion stability, intracellular signaling, and interact with the actin cytoskeleton. E-cadherins are the major component of AJs. They interact with the E-cadherins of adjacent cells and with the actin cytoskeleton [57,58]. Nectin–afadin complexes are also important as they form homophilic and heterophilic strands with adjacent cells [59,60].
3. Desmosomes, which are located at the most basolateral region of IECs. They are found in tissues that require mechanical forces. Indeed, they promote strong adhesive bonds between adjacent cells by connecting them to the intermediate filaments of the cytoskeleton. Its main constituents are cadherins, armadillo proteins, and plakins [61,62,63,64].

**Figure 3:**
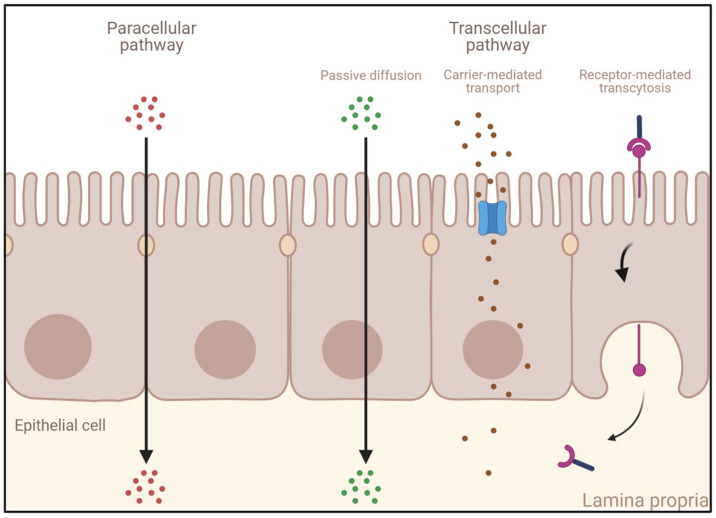
Different transport mechanisms across the intestinal epithelium. Created with Biorender.com (accessed on 24 January 2023).

A drug can cross the intestinal mucosa via several different mechanisms, depending upon its physicochemical properties. Small molecules and ions can use the paracellular pathway to cross the intestinal mucosa barrier. However, due to the intercellular junctions presented above, the transport of macromolecules can only be achieved across IECs. A transcellular transport can occur via several mechanisms (Figure 3):

- Passive diffusion: This process, which does not require energy expenditure, is a non-selective, non-saturable, and non-carrier-mediated transport through the phospholipid membranes of IECs driven by the concentration gradient of a compound [65]. Passive diffusion only allows small lipophilic molecules to diffuse at significant rates (e.g., gases such as O_2_ and CO_2_, hydrophobic compounds, small polar but uncharged molecules).
- Carrier-mediated transport: Large uncharged polar molecules (e.g., glucose) and charged molecules of any size (e.g., small ions such as H^+^, Na^+^, K^+^, and Cl^−^) are not able to cross the membrane by passive diffusion. Hence, they require specific transporters and channel proteins situated at the cell surface to be transported across the epithelium. This transport can be achieved either by a reversible facilitated diffusion, which, similarly to passive diffusion, induces a movement of solutes across membranes from the side of high concentration to the side of low concentration without energy (e.g., carbohydrates, amino acids, nucleosides, ions), or by an active and saturable passage against the concentration gradient requiring chemical energy (e.g., ATP hydrolysis) [66]. Other molecules are transported against the concentration gradient by using the electrochemical potential difference created by pumping ions out of the cell (e.g., the Na^+^-K^+^ pump).
- Endocytosis: This mechanism induces the internalization of extracellular molecules via several processes involving the formation of intracellular vesicles and not limited by the size of the cargo [67,68] such as pinocytosis, phagocytosis, or receptor-mediated endocytosis (RME) [69]. Pinocytosis is a fluid-phase endocytosis pathway that is non-specific and non-saturable (cellular incorporation of molecules in the extracellular fluid via micropinocytosis or macropinocytosis) [70], whereas phagocytosis is triggered by the binding to phagocytic receptors of particles larger than 0.5 μm in diameter (e.g., microorganisms, foreign substances, apoptotic cells). They are engulfed to form large intracellular vesicles called phagosomes that will fuse to lysosomes to create phagolysosomes and induce the degradation of the particles [71]. RME is an uptake mechanism occurring in different cell types and triggered by the binding of ligands on their specific membrane-bound receptors [69,72]. When followed by transcytosis, it enables their transport and is thus of high interest for the specific delivery of large molecules into the bloodstream.

#### 2.1.3. The Biochemical Barrier

One of the other challenges faced by any drug taken orally is the harsh environment within the stomach and the intestine that can cause many of the administered molecules to denature or degrade, resulting in a significant reduction in their effectiveness.

The stomach is the most challenging and acidic part of the GI tract because of the gastric juice, which has the role not only of initiating the digestion of food particles and converting them into chymus [73], but also to inactivate microorganisms in order to protect the GI tract from pathogens. The acidic pH, due to the presence of hydrochloric acid (around 1–2.5), and the activation of gastric enzymes (e.g., pepsin) in this environment represent the most important threats of the stomach [74].

In addition to the gastric enzymes, there are also pancreatic enzymes produced inside the pancreas and secreted into the intestinal lumen. They are activated by the increase in pH in the duodenum (pH = 4.0–5.5), the jejunum (pH = 5.5–7.0), the ileum (7.0–7.5), and the colon (can vary from 6 to 8 due to an important interindividual variability [47]) (e.g., aminopeptidases, serine proteases, carboxypeptidases, endopeptidases). These pancreatic enzymes are mainly abundant in the duodenum, and their concentrations considerably decrease along the GI tract [30]. Hence, some studies are trying to focus on a colon- or ileum-targeted drug delivery system to lower the risk of proteolytic degradation [75,76].

### 2.2. Challenges of the Oral Delivery of Biologics

Although the oral delivery of biologics is a highly desirable approach, owing to its convenience and patient compliance, this route of administration poses several challenges due to the barriers of the GI tract. First of all, biotherapeutics have to face the difficult conditions of the stomach and intestine. Indeed, recombinant biological molecules tend to have a lower gastric stability than common drugs because of their larger molecular weight and their complex structure that can be more easily disrupted by the threats of the stomach, such as the acidic pH and the activation of pepsin. The latter has a strong ability to degrade protein via the hydrolyzation of aromatic residues’ peptide bonds [74,77]. Then, biologics can be affected by the differences in pH values between the different segments of the GI tract caused by the intestinal fluid produced by the pancreas to preserve the IECs. On the one hand, it can lead to conformation alteration and precipitation by reaching the isoelectric points of the molecules [78], and on the other hand, the pancreatic digestive enzymes activated by this increase in pH can easily cleave biologics and thus impact their residential intestinal stability.

The first physical barriers encountered when a recombinant biological molecule reaches the intestinal lumen are the mucus/glycocalyx layers. Indeed, as described in the previous section, they hamper external large biotherapeutics from reaching the epithelium by acting like a size exclusion filter, decreasing the movement of entrapped macromolecules, which leads to the development of an aqueous unstirred layer [36]. The residence time of biologics is then reduced in the small intestine because of the constant turnover process of the adherent layers [37]. Underneath mucus and glycocalyx, the IECs are also a strong determinant of the poor permeability of biologics. Indeed, the transport of drug molecules through the mucosa depends on both their chemistry and their size. However, the important size of biotherapeutics impedes their paracellular transport, which is restricted to small drugs due to the intercellular junctions connecting the IECs [79]. Moreover, since biologics are large and unable to dissolve in the hydrophobic interior of the phospholipid bilayer [80], passive diffusion and carrier-mediated transport are also restricted for these biological drugs. Hence, endocytosis/transcytosis remains the mechanism of choice for transport across the intestinal mucosa. Nevertheless, to be able to cross the IECs in a specific manner, an RME is necessary [69,72].

In addition to the cited physical and biochemical barriers of the GI tract, other factors such as the gut microbiota [74,81] or mechanical damages (e.g., osmotic stress, peristalsis of the GI muscles) [30] can also impact the bioavailability of drugs and thus their therapeutic efficacy.

## 3. Models to Study Permeability of Biologics across the Intestinal Barrier

### 3.1. In Vitro Models

From simple monocultures to complex 3D models, different in vitro approaches have been developed to assess intestinal permeability [12]. Although each of these models has its limitations [82], some of them have proved to be useful as screening tools in the pharmaceutical field. The main advantages and limitations of the in vitro models presented are listed in Table 1.

#### 3.1.1. Caco-2 Cells

**Figure 4:**
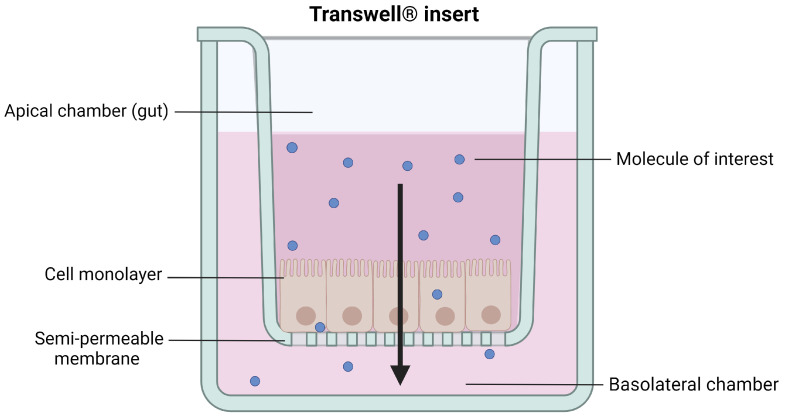
Schematic representation of cells grown on a Transwell^®^ insert. Created with Biorender.com (accessed on 17 January 2023).

Transwell^®^ inserts with a polycarbonate membrane are the most common systems used to recreate the intestinal interface in vitro They are composed of a filter with an uncoated semi-permeable membrane (0.4 µm pore), upon which the polarized monolayer is cultured. It separates the apical chamber corresponding to the intestinal lumen (donor compartment) and the basolateral chamber representing the blood vessels (receiver compartment) (Figure 4).

**Figure 5:**
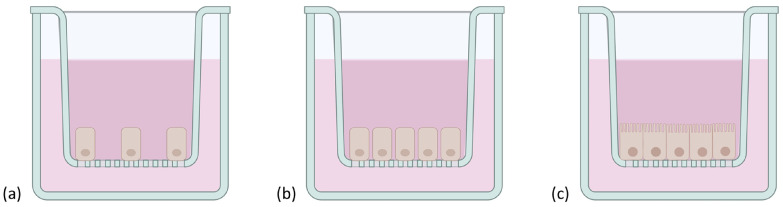
Schematic representation of Caco-2 cells grown on a Transwell^®^ insert. Cells were represented (**a**) at the beginning of the culture, (**b**), once confluent, and (**c**) structurally and functionally differentiated in mature enterocytes after 21 days. Created with Biorender.com (accessed on 17 January 2023).

However, obtaining primary cell cultures from intestinal tissues is still challenging, mostly because of the short life span of enterocytes, the dependency of cells on the extracellular matrix, and their incapacity to form an organized polarized monolayer of epithelial cells [83]. To overcome these limitations, immortalized continuously growing (tumor) cell cultures on inserts have been extensively studied [84]. Indeed, the most widely used in vitro intestinal monoculture model, the Caco-2 cells, is based on a human colorectal adenocarcinoma. This model enables a confluent monolayer to be obtained, which spontaneously differentiates, structurally and functionally, in mature enterocytes 21 days after seeding on Transwell^®^ inserts (Figure 5). The polarized cells, with an apical microvilli network and a basolateral surface, are connected to each other by TJs [85] and express brush border enzymes [86], membrane receptors, carrier transport systems [87,88], and some efflux proteins such as the functional P-glycoprotein [89]. The integrity of the model can be assessed via the measurement of transepithelial electrical resistance (TEER) reflecting the ionic conductance of the paracellular pathway of the monolayer [90,91] and via the evaluation of the passive permeation of different molecules used as permeability markers to certify the tightness of the monolayer [92,93].

Caco-2 cells have already been used to assess the transport of many drugs. Several permeability data obtained with this model even showed a strong correlation with in vivo results in humans, in particular for passive transport [94,95]. This simple and low-cost approach has also been used to study the active transport of macromolecules such as insulin [96,97], vitamin B12 [98], or transferrin [99].

Even if Caco-2 cells became a valuable tool to investigate the permeability of small conventional drugs and recombinant biological molecules, they also have limitations. First, this monoculture only expresses one type of cell. Thus, the model lacks the complexity found in intestinal human tissues and does not produce mucus [100]. Then, due to their tumoral origin, Caco-2 cells might display different characteristics and deviate from an intestinal mucosa made of non-cancerous cells. Caco-2 cells have also been criticized because of their heterogeneity [101], the variability of the results between laboratories [102], and because of their higher tightness compared to human or animal small intestines [103,104]. Finally, Caco-2 cells require 21 days of growing to be fully differentiated, which limits the throughput of this approach. To overcome some of these issues, several studies have focused on decreasing the time of culture by modulating experimental conditions [105,106,107], and several subclones of Caco-2 cells have been isolated and studied to obtain more homogeneous populations [108,109,110].

In summary, although this model is simple, widely used, cheap, commercially available, and the data obtained with this approach are very informative for a preliminary screening, complementary studies using more complex models are needed.

#### 3.1.2. MDCK Cells

Mardin–Darby canine kidney (MDCK) cells have been considered as an alternative to the Caco-2 cells model to assess drug permeability [111,112]. Indeed, they are able to differentiate into columnar polarized cells with brush borders and TJs faster than Caco-2 cells (only 3 to 5 days of culture after reaching confluence). This quicker culture is a real advantage as it enables an increase in the throughput of a permeability screening and the shortening of labor in case of contaminations. Moreover, compared to Caco-2 cells, the MDCK-I strain has a TEER value closer to the in vivo small intestine [84]. However, although MDCK cells have already been used to assess the transcellular transport of recombinant biological molecules such as insulin [113], transferrin [114], Immunoglobulin G (IgG) [115], or peptides targeting the neonatal Fc receptor (FcRn) [116], this cell line is mostly used to evaluate passive absorption mechanisms. Indeed, contrary to Caco-2 cells derived from human carcinoma cells, MDCK cells have canine and renal origins, which leads to a lower expression of some transporters or receptors, and a weaker metabolic activity. The heterogeneity of MDCK cells can also lead to variable results [117] and, like the Caco-2 model, they do not mimic the multicellular in vivo epithelium. Thus, despite its suitability for a rapid and high-throughput preliminary study, the origin of the model and the lack of complexity do not reproduce accurately what occurs in vivo in humans.

#### 3.1.3. Caco-2/HT29 Cells

To overcome some of the previous model limitations such as a better reproduction of the multicellular intestinal epithelium, co-cultures and tri-cultures of different cell types have been imagined. For instance, a co-culture of Caco-2 and mucus-secreting HT-29 cells has been developed to generate a more predictable experimental cell model with a TEER value closer to the human intestine in vivo [91].

HT-29 cells, which derive from human colorectal adenocarcinoma cells, were initially used to investigate the role of mucus on drug transport across the intestinal epithelium. Indeed, after 21 days post-confluence, and when cultured in a medium containing methotrexate, these cells differentiate into mature polarized goblet cells that express both secretory and membrane-bound mucin types. However, unlike the Caco-2 cells model, HT-29 cells are unable to generate proper TJs [118]. Hence, the combination of the two cell types results in a co-culture model that more closely mimics the human intestinal conditions by maintaining the differentiated features of both models, such as the expression of a mucus layer and loose TJs closer to those of the small intestine in vivo [119]. This approach has already been used to assess the transport of native insulin and insulin/transferrin conjugates with a more accurate depiction of overall permeability that took into account the effect of diffusion through the mucus [120]. However, despite the obvious advantages, one of the major drawbacks of this co-culture remains the non-uniformity of the mucus layer coverage [11]. The permeability across the monolayer can also be different depending on culture conditions (e.g., seeding ratio, cell culture time, the medium [121]), leading to a high variability of results.

#### 3.1.4. Caco-2/HT29/Raji B Cells

**Figure 6:**
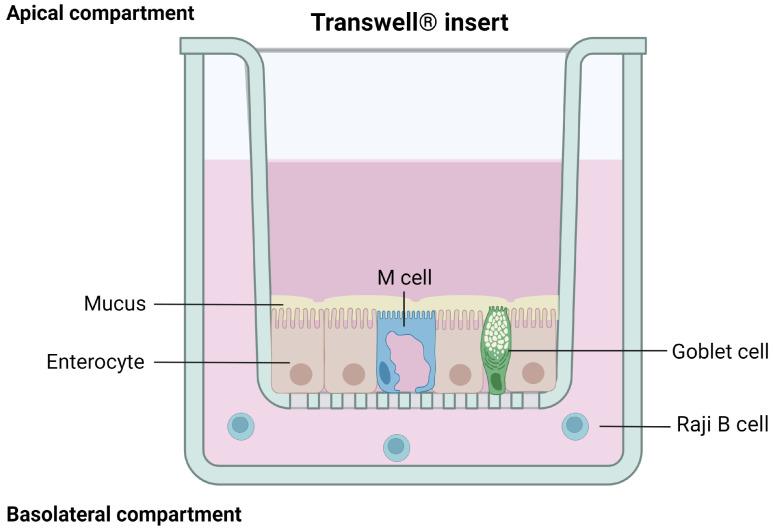
Schematic representation of a well-differentiated Caco-2/HT-29/Raji B cells model. Created with Biorender.com (accessed on 12 February 2023).

M cells have a unique morphology and are used by several microorganisms, microparticles, and antigens to cross the epithelial barrier [122,123]. Hence, this cell type is important to obtain a more representative model of the human intestinal epithelium. By co-cultivating Caco-2 cells with murine Raji B lymphocytes, without contact and over 4 to 6 days, it is indeed possible to obtain M cell morphology from the cancerous cells [124,125]. Although this approach does not entirely mimic the intestinal epithelial layer, it opened up the possibility to build a more representative model of the intestinal barrier by setting up a triple culture of Caco-2/HT-29 (respectively 90/10% on an insert) [126] and Raji B cells (added to the basolateral chamber after 14 days) [127]. The characterization of this triple culture model confirmed that Caco-2 cells representing the mature absorptive enterocytes are expressed, as well as HT-29 cells producing the mucus layer, and finally Raji B cells inducing M cells’ phenotype. The tightness of the model is also closer to physiological tissue, with TEER values similar to the ones obtained with Caco-2/HT29 co-cultures (around 200 Ω·cm^2^ after 21 days of culture [126]) (Figure 6).

This model has already been used to successfully assess the intestinal permeability of drugs, including biologics such as insulin (in solution or in nanocarriers) [128,129], and data obtained showed that this technique is efficient at predicting the intestinal permeability of proteins when compared to results from ex vivo assays [128]. Hence, this approach seems promising to develop a more physiologically and functionally relevant in vitro model that would allow for the evaluation of the intestinal permeability of recombinant biological molecules in a small-scale size range. However, the set-up of this co-culture is complex, which involves a high variability of results between laboratories. Moreover, it still leans on Caco-2 cells and thus shares some of their drawbacks.

#### 3.1.5. 3D Culture Models

3D culture models based on extracellular matrixes have been developed in order to address the lack of representation of the intricate network of biochemical signals of the 2D monolayers. These systems allow researchers to mimic key factors of tissues and are much more representative of the in vivo environment [130].

##### EpiIntestinal

EpiIntestinal, a 3D in vitro intestinal tissue model using primary cells with a human intestinal origin, has been developed by MatTek (Ashland, MA, USA), to evaluate toxicity, metabolism, drug absorption, and drug efficacy [131,132]. It incorporates different IECs (enterocytes, Paneth cells, M cells, tuft cells, stem cells) into a highly differentiated polarized epithelium mimicking the human structure and physiology. It displays brush borders at the apical side, functional TJs, and the presence of mucus-secreting granules. This model is cultured at the air–liquid interface on cell culture inserts (with 24- or 96-well screening plates [133]) to enable physiological exposure conditions, and can be kept in culture for up to a month. A variation of this model includes the underlying lamina propria.

This model was proved to be more relevant to evaluate drug absorption and metabolism in the human GI tract than Caco-2 cells [134,135]. It has already demonstrated its ability to study an active transport of the cholix protein by receptor-mediated transcytosis (RMT) [136,137]. Thus, this approach seems very promising to accurately predict the permeability of biologics across the intestinal epithelium. However, to date, no other data using this model to assess the transport of macromolecules have been published. As a commercial product, the costs of such a tool can also be a limitation.

##### Gut-on-a-Chip

**Figure 7:**
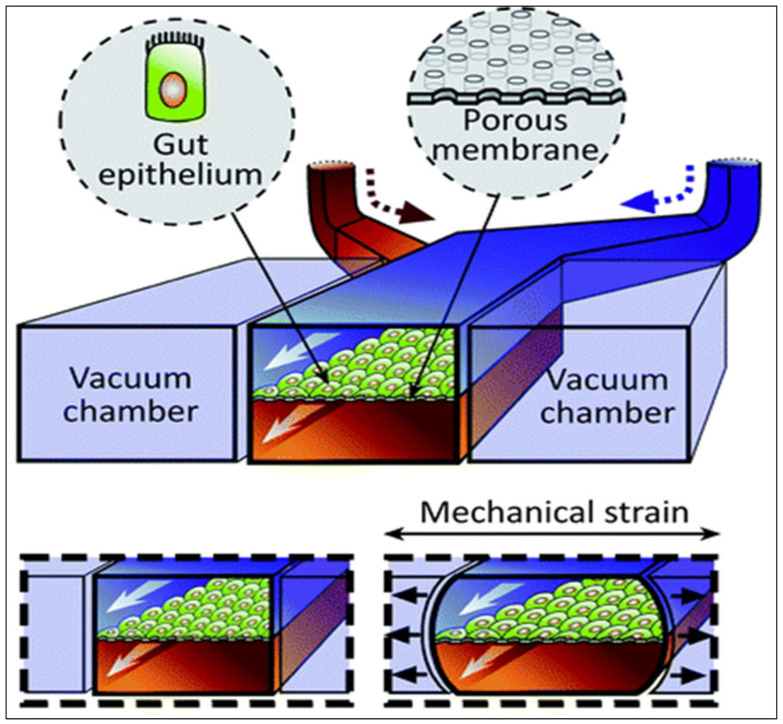
Schematic representation of the gut-on-a-chip device. Reproduced from [144] with permission from the Royal Society of Chemistry.

A 3D method called “gut-on-a-chip” has been developed in order to better mimic the structure of the intestine, its physiology, and its transport abilities [138,139,140]. It uses a microfluidic device containing a thick, porous membrane coated with an extracellular matrix for cell culture (e.g., Caco-2 cells), surrounded by upper and lower microchannels simulating the intestinal lumen and the blood circulation (Figure 7). Moreover, vacuum chambers, located at both sides of the device, regulate the laminar fluid flow through microchannels and imitate digestive shear forces, such as peristalsis. These simulated muscle contractions allow the better representation of in vivo transport by inducing an increase in the permeability without any modification to the model’s tightness [141,142].

Unlike static culture systems, this technique with dynamic conditions develops a fully matured monolayer in only few days. Indeed, the fluid flow associated with the shear stress promotes 3D intestinal villi, basal crypt formation, and the development of four different intestinal cell types (157). To have a better representation of the intestinal physiology, bacteria isolated from the human gut can also be cultured on top of the cells [91,143].

With the numerous simulated physical and functional features of in vivo intestine, this system can be used for transport screening and toxicology studies of new therapeutic molecules [144,145,146]. Several variations of this device are also being developed [147,148] in order to generate intestinal disease models that would allow for the assessment of the transport of a molecule through a pathological intestinal barrier [149,150]. However, although the promising “gut-on-a chip” model is extensively studied and constantly evolving, it has not yet been used to predict the intestinal transport of biologics.

##### Intestinal Organoids

Since the monolayer of the intestinal epithelium is renewed every 4–5 days while maintaining a continuous active mucosal barrier thanks to stable intestinal stem cells [151], Sato et al. have successfully developed a method to generate villi-like epithelial domains with all types of IECs by isolating mice intestinal crypts and establishing long-term culture conditions of Lgr5+ stem cells [152]. Following this discovery, a promising 3D in vitro culture model, having similar organ functionalities to the tissue of origin, has been imagined: intestinal organoids, or mini-guts [153].

**Figure 8:**
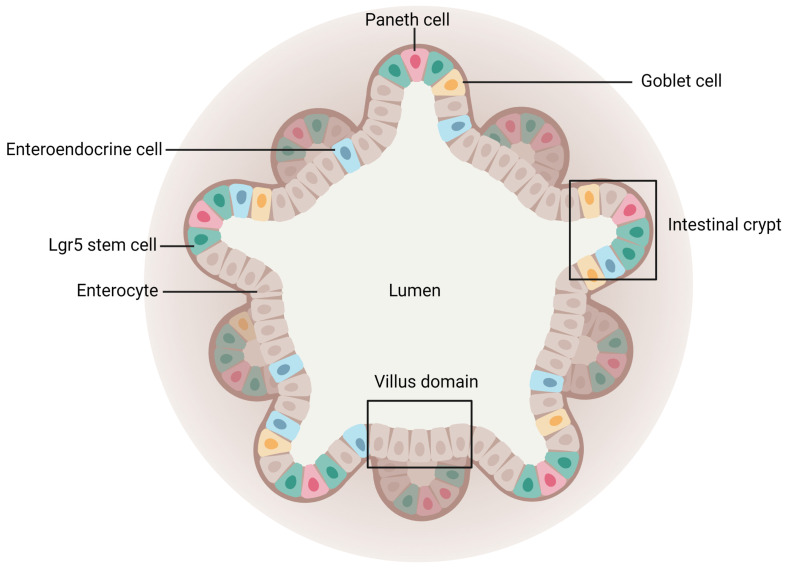
Schematic representation of an intestinal organoid. Created with Biorender.com (accessed on 21 March 2023).

Intestinal organoids derive either from Lgr5+ stem cells or from the isolation of intestinal crypts recovered from humans or animal species embedded in Matrigel [154]. To develop intestinal organoids, the medium used is supplemented with key niche signals, such as epidermal growth factor to promote cell proliferation, R-spondin 1 to maintain the stem cell population, or Noggin necessary for passage as it limits differentiation. Following stem cell proliferation and differentiation, the mini-gut model can mimic the morphology and physiology of in vivo tissue. Indeed, intestinal organoids form a spherical 3D structure with crypt-like domains (stem cells, Paneth cells) and villi-like regions displaying all other intestinal mature cell types. Both surround the central lumen (Figure 8) containing dead cells from the renewed epithelium [83,155]. This model also presents other important features of the intestine, such as metabolizing activities or mucus secretion [156].

Intestinal organoids better reproduce the intestinal mucosal barrier than conventional 2D approaches [157]. Moreover, they remain very close to freshly isolated small-intestinal crypts after several months of culture, freezing, and replating. Hence, their long-term culture is an advantage [158]. However, intestinal organoids cannot imitate biomechanical forces encountered by stem cells in vivo, and they require the use of a not-well-defined three-dimensional matrix, the Matrigel, which can result in the variability of data [159]. These models are also heterogeneous in terms of their viability, size, and shape, and their 3D spherical geometry is unsuitable for relevant drug transport studies [153]. To overcome this latter drawback, microinjections and trituration have been considered but these strategies are costly, time-consuming, and can alter the tissue [160,161]. Hence, another method consisting of a 2D self-renewing monolayer development that derives from an organoid has been investigated. This technique enables researchers to obtain a model expressing all IECs and with an easier conformation, which allows access to both apical and basal sides to assess the permeability of compounds [162,163,164,165].

**Table 1:** Advantages and limitations of the different in vitro models.

Model	Advantages	Limitations	References
Caco-2 cells	Higher accuracy in replicating the enterocytic phenotype Polarized monolayer with TJs Low cost Extensively studied Commercially available	Cancerous origin Time-consuming Single cell-type and lack of physiological factors (e.g., mucus) Absence of dynamic conditions	[85,101,102,104]
MDCK cells	Polarized monolayer with TJs TEER similar to human physiology Short culture time Commercially available	Canine and renal origins Single cell-type and lack of physiological factors (e.g., mucus) High variability Absence of dynamic conditions	[84,111,117,166]
Caco-2/HT29 cells	Two types of IECs Presence of mucus Closer TEER to small intestine in vivo than Caco-2 cells	Cancerous origin Time-consuming Non-uniformity of mucus layer coverage High variability depending on culture conditions Absence of dynamic conditions	[11,118,121]
Caco-2/HT29/RajiB cells	Three types of IECs Presence of mucus More reliable for active transport Closer TEER to small intestine in vivo than Caco-2 cells	Complexity of the model Time-consuming High variability Absence of dynamic conditions	[126,127]
EpiIntestinal	Easy to use Presence of different IECs Presence of mucus High reproducibility High throughput TEER similar to human physiology No culture time needed	Costly Absence of dynamic conditions Only a few pieces of data published for transport of biologics	[131,132,134,135,166]
Gut-on-a-chip	Reproduction of the 3D typology with crypts, villi, and different IECs Presence of mucus Dynamic (shear forces) Short culture time Flexible control of system parameters (fluid flow, oxygen concentration) Possible to simulate intestinal diseases	Complex model Need for microfluidic skills No data published for transport of biologics	[138,140,141]
Intestinal organoids	Reproduction of the villus–crypt morphology of the epithelium with all IECs Presence of mucus Can be expanded indefinitely Can be grown 2D or 3D	Complex model Geometry unsuitable for transport assays Absence of dynamic conditions High variability of the model (e.g., shape, size) Need for primary cells of intestinal crypts	[155,156,158]

### 3.2. Ex Vivo Models

Ex vivo models are based on living and functional extracted tissues cultured in a controlled external environment mimicking physiological conditions. They represent an interesting compromise between in vitro and in vivo experimental models since they display a higher complexity than cell-based models and allow the study of permeability on different segments of the GI tract to consider regional differences characterizing site-specific absorption capabilities (e.g., pH, proteolytic activity, mucus thickness, surface area) [13]. Hence, several systems have been developed and each of them possesses singular advantages and limitations (Table 2). For instance, ex vivo models can better reproduce the characteristics of the intestinal mucosa by mimicking more closely what occurs in vivo, but they usually have a lower throughput, making them less suitable in the early phase of a pharmaceutical screening. For these reasons, these techniques are generally used to complement and confirm the selection made using cell-based techniques in a more complex system.

#### 3.2.1. Everted Intestinal Sacs

Everted intestinal sacs, a method first described with rat and hamster intestines in 1954 for glucose and amino acid transport studies [167], is nowadays considered for several applications (e.g., active drug/recombinant biological molecule absorption and interaction studies, pharmacokinetics, efflux transport investigations). This model is based on fresh intestine (removed from an anesthetized or a dead animal) cut into sections of 2 to 4 cm, washed, and everted on a rod or a tube. With this approach, the serosa becomes the inside of the sac, whereas the mucosal surface faces the outside. Then, to set up the model, sacs are tied at one end, filled with buffer with a blunt needle, closed at the other end, and immersed in a container with an oxygenated solution at 37 °C helping to keep the tissue viable [11] and containing the molecule to test [14].

This model is advantageous for multiple reasons: it is simple, fast, not expensive, one animal is sufficient to test numerous drugs simultaneously, and it requires limited quantities of molecules, which is a particularly important aspect for biologics [168]. Thus, this method is very interesting to easily and quickly study the transport of recombinant biological molecules across the intestinal epithelium. However, as demonstrated by histological studies assessing tissue morphology variations over time, this model also rapidly and gradually loses structural integrity. Structural changes can be noticed after only 5 min of incubation and a complete disruption of the epithelium is observed after 1 h [169,170]. Hence, in order to preserve the integrity of the tissue, which is not monitored during the experiment, the preparation of the experiment has to be quick and precise, and the incubation time has to be short [171].

In order to overcome some of the limitations, several variations of this technique have been developed over the years. For instance, to be able to recover samples from the serosal side at several time points during the experiment, the tissue can be cannulated with polyethylene tubing [172,173]. Although it is not a common practice for everted sacs, it is also possible to remove the serosa and both longitudinal and circular smooth muscles from the intestinal sections, which can lead to uptake or transport underestimation and decrease the viability of the intestine [174,175]. Finally, a non-everted intestinal sac method can be used as an alternative in order to avoid damages to the tissue during eversion. With this technique, preparation is easier and sampling can be performed with fewer difficulties [176]. Nevertheless, the permeability of actively transported molecules seems to be higher when the sacs are everted [177].

#### 3.2.2. Diffusion Chambers

##### Ussing Chambers

**Figure 9:**
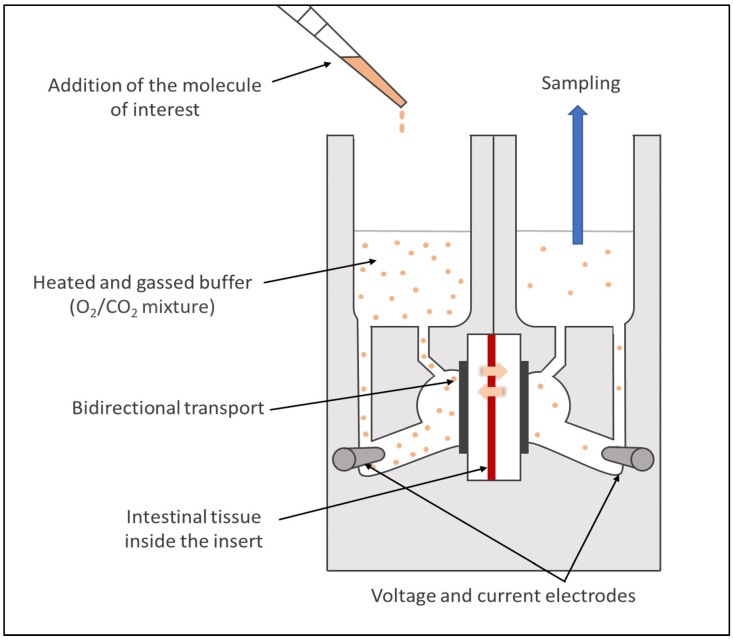
Schematic illustration of an Ussing chamber.

The Ussing chamber system, first introduced in 1951 by Ussing and Zehran [178], became a widely used model to assess drug permeability. For this approach, a viable intestinal segment recovered from a fresh intestine, and without a serosal layer and muscle tissues, is mounted into sliders situated vertically between the two acrylic compartments of the chamber. One of the compartments represents the mucosal surface (luminal compartment), where the molecule of interest is diluted in buffer heated at the animal body temperature, and the other simulates the serosal side (basal compartment) with an equivalent volume of the same buffer, free of the molecule (Figure 9). Although less common, the molecule can also be exposed at the serosal level [179] as this model allows researchers to study bidirectional transport across the intestinal epithelium.

Each compartment of the chambers has an air/gas inlet to oxygenate the intestinal segments with a 95% O_2_/5% CO_2_ mixture. It enables researchers to keep the tissue viable, to create a fluid movement with rising bubbles, and thus reduce the unstirred water layer [180]. The system is also heated with a heater block base regulating the temperature via an external thermal water circulator. The integrity of the tissue is continuously monitored during the ex vivo assays thanks to electrodes connected to each chamber measuring the TEER [100].

This system can be used to evaluate the permeability of small molecule drugs and biologics [68,181] across epithelial tissues from both humans [13,182] and animals (e.g., rats, rabbits, pigs [183,184,185]), on different regional segments of the intestine, and with different physiological conditions (e.g., use of simulated media, different pH). However, although this approach is a relevant strategy to assess the active transport of recombinant biological molecules across viable tissues, it requires a time-consuming preparation that limits the throughput. In addition, the tissue can be damaged during the preparation and/or the mounting stages. Finally, similarly to all the ex vivo techniques, several factors can lead to an important variability of the results (e.g., experimental conditions, animals).

##### Franz Cells

**Figure 10:**
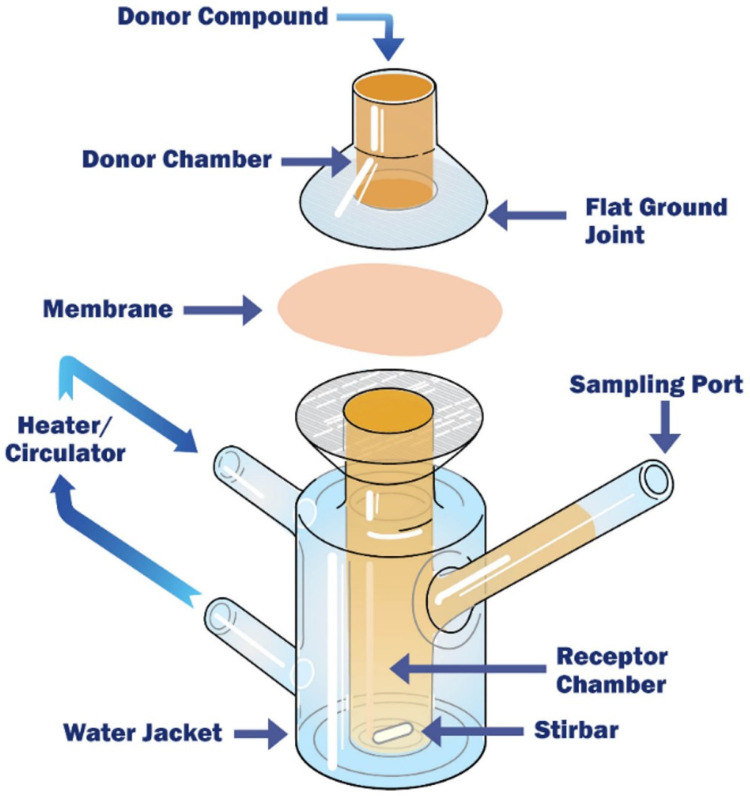
Schematic illustration of a Franz diffusion cell. Reproduced from [190] with permission from Permegear.

Franz diffusion cells, one of the widely used model to study skin permeation for transdermal drug administration [186,187], is also an approach considered more and more as an intestinal permeability tool. Indeed, similarly to the Ussing chamber system, Franz diffusion cells have two temperature-controlled compartments separated by a fresh human or animal intestinal segment (usually without the underlying serosa and muscle layers): the donor compartment with agitation performed by gas bubbling, and the receptor chamber where a magnetic stirrer adequately homogenizes the solution [188,189] (Figure 10). Contrary to the Ussing chambers having an equal volume in their two compartments, Franz diffusion cells have donor chambers filled with a smaller volume than the receptor ones. However, sampling can also be easily performed at different time intervals during the experiments.

One of the other main differences with the Ussing chamber system is the orientation of the tissue, which is mounted horizontally in Franz diffusion cells andenables a direct contact between the molecule tested and the intestinal epithelium. Moreover, the integrity of the tissue is not monitored continuously during Franz diffusion cell experiments. The TEER is generally measured only at the beginning and at the end of the assays. Hence, to have a better assessment of the integrity of the intestinal epithelium, permeability markers are often used in addition to the TEER [188].

Concerning the limitations of this model, Franz diffusion cells share similar challenges to the Ussing chamber system. It is also a model which, to date, has been mainly focused on the transport of small molecule drugs [188,191,192].

#### 3.2.3. InTESTine™

**Figure 11:**
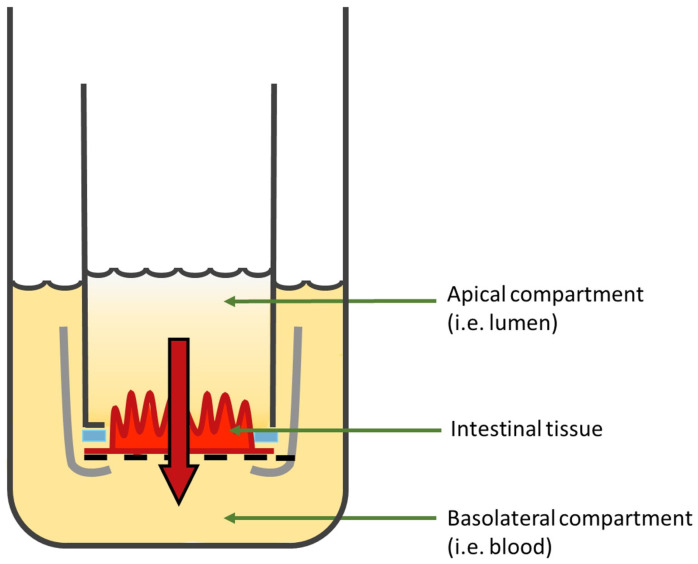
Schematic illustration of one chamber of the InTESTine™ model. Figure was adapted from [194].

In order to overcome the complexity and the low throughput of diffusion chambers, a commercially available model allowing 24 or 96 simultaneous incubations per system on viable and healthy segments from a porcine GI tract (without the outer muscle layers) has been developed by the Netherlands organization for applied scientific research (TNO) and is called InTESTine™ (Figure 11). This two-compartment model has an easy set up and can be incubated at 37 °C in a humidified oxygenated incubator and on a rocket platform to mimic the body’s conditions, reduce the unstirred water layer, and avoid evaporation. This approach can be used to assess the intestinal permeability of a molecule in different intestinal regions, with a mucus layer, in the absence or presence of a microbiota, and with or without simulated fluids [193]. In addition, non-specific binding and contamination risks are reduced by the fact that the device is composed of disposable glass materials.

The InTESTine™ model is advantageous compared to diffusion chambers in terms of throughput. It also has a similar intestinal viability as demonstrated by TEER measurements and the passage of a permeability marker [193]. Hence, InTESTine™ seems to be a promising system for ex vivo permeability investigations across the intestinal barrier. A recent study has even demonstrated the ability to apply human intestinal tissues into an InTESTine™ device suitable for a standard 6- or 24-well plate format [195]. However, it must be considered that the cost for this device is high and no macromolecule transport data have been published yet.

**Table 2:** Advantages and limitations of the different ex vivo models.

Model	Advantages	Limitations	References
Everted intestinal sacs	Simple and fast set-up No need for a particular system Lot of samples from one animal Use of small number of molecules of interest Possible sampling by cannulation with polyethylene tubing	Serosa and muscles layers commonly not removed (underestimation of uptake/transport, decrease in viability) Complete loss of structural integrity after 1 h Potential damages during eversion No monitoring of the viability during the experiments	[14,168,169,171]
Ussing chambers	Several samples from one animal Serosa and muscles layers removed Possible studies in different physiological conditions Continuous monitoring of the viability Easy sampling Reduced unstirred layer via gas bubbling	Require an expensive Ussing chambers system Time-consuming preparation steps Low throughput Viability of approximately 2 h Potential damages during the tissue preparation	[14,180,196]
Franz diffusion cells	Several samples from one animal Serosa and muscles layers removed Possible studies in different physiological conditions Reduced unstirred layer via magnetic agitation and gas bubbling Easy sampling	Require Franz diffusion cells Time-consuming preparation steps Low throughput Viability of approximatively 2 h Potential damages during the tissue preparation Viability not monitored continuously Mostly used, to date, for small molecule drug transport	[188,189,191,197]
IntesTINE^TM^	Easy to use Not time-consuming High throughput Possible studies in different physiological conditions Agitation possible in an incubator to avoid unstirred layer formation and evaporation Lower risks of contamination (disposable glass materials) Commercially available Development of a device with human intestine	Costly Viability of 2 h Viability not monitored continuously No data published yet with biological molecules	[193,195]

## 4. Strategies to Improve the Oral Delivery of Biologics

### 4.1. Strategies to Increase Stability

Most recombinant biological molecules are highly sensitive to the physiological barriers of the GI tract (i.e., mainly the low pH and the presence of digestive enzymes). Thus, in order to improve the oral delivery of biotherapeutics, different approaches have been developed to provide protection and allow them to reach the intestinal mucosa [31]. The most commonly used ones, as well as some promising new strategies, are described in this section.

#### 4.1.1. Enteric Coating

The acidic pH of the stomach and pepsin degradation threatening the stability of recombinant biological molecules can be easily overcome with the use of an enteric coating. It is a formulation that allows the dissolution of the coating of a tablet, capsule, or particles containing the drugs in the higher pH of the small intestine, thanks to the deprotonation of weakly acidic functional groups of a pH-responsible polymer (e.g., cellulose acetate phthalate, methacrylic acid copolymers) [198]. Most of the products already developed for protein or peptide oral administration use this strategy to bypass the stomach environment (e.g., oral insulin of Diabetology Ltd. (London, UK/Perth, Australia) which completed phase II clinical trial; oral insulin of Oramed Pharmaceuticals Inc. (New York, NY, USA) that reaches clinical trial phase III [27,199]; or the marketed MYCAPSSA^®^ of Chiasma pharmaceuticals (Needham, MA, USA) for the delivery of octreotide [27,28,199]). Nevertheless, the enteric coating alone is not sufficient to improve the stability of a drug and protect it from enzymatic degradation. Indeed, it only exerts its activity in the stomach and not in the small intestine, where multiple pancreatic enzymes are also present. Hence, enteric coating must be used in combination with other approaches protecting the molecules of interest from the biochemical barrier [77].

#### 4.1.2. pH Modulators

Various digestive enzymes threatening biologics’ stability are active at a specific pH. For example, stomach pepsin exerts its effect only at a pH lower than 3 [200], whereas pancreatic enzymes are dependent on the pH increase in the small intestine. Hence, the modulation of the microenvironment’s pH via proteolytic enzyme inhibition was considered in several studies to protect recombinant biological molecules. For instance, enteric-coated formulations containing salmon calcitonin and various amounts of citric acid were tethered to a Heidelberg capsule, a high-frequency micro-electronic transmitter allowing continuous inter-abdominal pH monitoring, and given orally to beagle dogs [201]. After oral administration, blood samples were regularly collected. Results obtained have shown that the highest concentrations of salmon calcitonin in the systemic circulation were always associated with the lowest small intestinal pH values induced by the more important amounts of citric acid in the formulations. These data proved that the oral absorption properties of this model peptide drug could be modulated by changing intestinal pH. Indeed, salmon calcitonin is a substrate for the pancreatic serine protease trypsin, whose maximal activity is around pH 5 to 6. Hence, reducing intestinal pH probably stabilized the peptide in the GI tract and allowed a better absorption of the intact salmon calcitonin.

A double-blind phase III trial was also completed with success for the treatment of postmenopausal osteoporosis, using an oral delayed-release formulation of salmon calcitonin (ORACAL^®^ by Tarsa Therapeutics, Philadelphia, PA, USA) inserted in an enteric-coated capsule formulated with citric acid [202]. This study demonstrated the positive safety and efficacy of this strategy. However, it is important to ensure that the pH decrease does not prevent the enteric coating dissolution in the small intestine if both approaches are used in combination. Other agents modulating the pH have been studied such as fumaric acid, itaconic acid, or tartaric acid [203,204].

#### 4.1.3. Enzyme Inhibitors

To protect recombinant biological molecules from proteolytic degradation, a strategy based on the inactivation of the enzymatic barrier using inhibitors has been studied. These inhibitors usually bind reversibly or irreversibly to the specific site of the targeted enzyme to reduce or inactivate its activity [205]. Multiple types of inhibitors can be used depending on the enzyme to inactivate, such as non-amino acids, amino acids and modified amino acids, or peptides and modified peptides [206]. Non-amino acids (e.g., small molecules chemically synthetized) and amino acids are highly toxic and are thus avoided as they are absorbed faster than the therapeutic molecules given their low mass [22]. Peptides and modified peptides, the most used to inactivate enzymes, include inhibitors of serine proteases [207,208,209] or pancreatic endopeptidases [210]. For instance, the degradation of insulin in the presence of α-chymotrypsin has been slowed down by duck ovomucoids, and the flux of insulin across rat jejunum mounted in Ussing chambers was improved [26].

However, despite several encouraging studies, the co-administration of biologics with enzyme inhibitors is still being discussed. Indeed, they are often used in long-term therapies and inhibitor activity may be weakened by several factors such as rapid dilution, digestion, etc. Thus, they have to be used at high concentrations, which could lead to side effects such as the absorption of unwanted molecules or damages to the epithelium tract. It could also induce a disturbed digestion of nutritive compounds due to a deficiency of pancreatic enzymes [211], as well as the increase in enzyme secretion due to feedback regulation [212,213,214,215].

To overcome these issues, protease inhibitors can be combined with other strategies used to enhance oral delivery. The co-administration of specific enzyme inhibitors with permeation enhancers (PEs, presented in Section 4.4.3) has particularly been investigated and some of the candidates have even reached clinical trials. For example, Oramed Pharmaceuticals Inc. has developed a protein oral delivery (POD™) technology [199] that led to a phase III clinical trial for insulin oral delivery. They used a three-pronged strategy composed of the active protein inserted in a pH-sensitive capsule promoting small intestinal release, soybean as a protease inhibitor, and ethylenediaminetetraacetic acid (EDTA) as a chelating agent to increase paracellular permeability [27]. Dexcel Pharma Technologies Ltd. (Or Akiva, Israel) also worked on insulin with the hypothesis that releasing the inhibitor and the PEs before the active protein might generate a more favorable environment for the molecule of interest. Hence, they developed ChronotropicTM platform technology using a two-pulse colonic release of insulin with camostat mesylate as protease inhibitor and sodium glycocholate as an absorption enhancer. This system has demonstrated its ability to improve the in vitro bioavailability of the recombinant biological molecule [216].

### 4.2. Strategies to Penetrate the Mucus Layer

Since biologics have to cross the intestinal mucus layer in order to reach the IECs, mucus-penetrating systems have been investigated. The first idea was to use mucolytics, drugs generally employed to clear mucus from lungs affected by various respiratory conditions [217]. However, their repeated use could induce injuries to the intestinal epithelium. Therefore, another approach has been extensively studied to penetrate deeper and faster into the mucus layer: the use of nanoparticles (NPs, presented Section 4.4.1). Inspired by viruses [218], scientists have indeed identified some optimal parameters for that purpose, such as a small size (30–200 nm) [219], a highly hydrophilic nature, the absence of mucoadhesive hydrophobic areas, and a densely charged yet net neutral surface [220]. To increase the hydrophilicity of the NPs’ surface, polymeric particles are often coated with polyethylene glycol (PEG) or other polymers (e.g., polyvinyl alcohol and N-(2-hydroxypropyl)methacrylamide copolymer (pHPMA)) [34,221]. This modification usually turns unmodified mucoadhesive NPs into mucus-penetrating polymeric particles without mucoadhesive properties. The particle’s shape also plays a role in the mucus-penetrating capacity [222]. For example, nanorods penetrate into the mucus more efficiently than spherical NPs with the same chemistry, as they rotate within the mucus and extend their residence time in the mucosa [223].

The mucus-penetrating strategy is often used in association with other approaches. For instance, a successful study using NPs coated with hydrophilic pHPMA encapsulating insulin and cell-penetrating peptides (CPPs, presented in the section “Cell-Penetrating Peptides”) has demonstrated an excellent NP penetration in mucus. With this strategy, the encapsulated protein was absorbed 20 times more than free insulin in vitro. It also elicited a significant hypoglycemic response in diabetic rats [224].

### 4.3. Strategies to Increase Contact Time with the Epithelium and Induce a Site-Specific Release

#### 4.3.1. Thiolated Polymers

Oral mucoadhesive delivery systems are usually based on mucoadhesive polymers that delay the transit time of drugs through the intestine by their bioadhesion to the mucin-epithelial cell surfaces [225]. For example, thiolated polymers, also called thiomers, are polymers that contain thiol groups on their polymeric backbone. They are used in pharmaceutical formulations to enhance drug delivery by increasing the contact time of biologics to the absorptive epithelial cells, thanks to the formation of disulfide bonds with the cysteine-rich subdomains of mucins, the major component of mucus [226,227]. The thiol groups can also disrupt the structure and allow the biotherapeutics to diffuse more easily through the mucus and into the bloodstream. Furthermore, thiomers can protect recombinant biological molecules from degradation via proteolytic enzymes in the gut. Indeed, the carboxylic acid groups on the thiomers can form electrostatic interactions with the positively charged enzymes, preventing them from breaking down the biologics. Thiolation can be realized on multifunctional polymers such as chitosan [228], polycarbophil [229], or poly(acrylic acid) [230], which have different abilities (e.g., enhancement of the permeation, prevention of enzymatic degradation) [231]. Their strong adhesion induced by the thiolation allows them to exert their effects in close contact with the intestinal epithelium.

Thiomers are already used to improve the delivery of small molecule drugs but their application to biologics is still under investigation. However, promising results have already been obtained. For instance, trimethyl chitosan-NPs promoted a higher Caco-2 cell internalization and a two-fold increase in encapsulated insulin transport through rat intestines compared to unmodified NPs [232].Hence, these polymers represent a promising strategy to improve the oral administration of biotherapeutics, which can be encapsulated within thiomers or attached to them via covalent bonding. However, thiolated polymers are prone to oxidation at a physiological pH. Thus, thiol groups have to be protected by a specific formulation.

#### 4.3.2. Intestinal Patches

Mucoadhesive intestinal patches were inspired by transdermal patches. They adhere to the mucus, create a high concentration gradient, and increase the residence time of biologics to promote their intestinal transport [233,234]. These devices also prevent macromolecule degradation. Intestinal patches are most of the time composed of three layers with

- a pH-sensitive layer generally made of materials with pH-dependent solubility (e.g., Eudragit^®^ polymers L or S [235]) to bypass the acidic environment of the stomach and avoid drug delivery before reaching the localized site in the small intestine;
- a mucoadhesive/drug reservoir layer with the molecule of interest (dissolved, suspended, or incorporated as microspheres into the layer); the mucoadhesive components (e.g., chitosan, thiomers); and other excipients (e.g., PEs, enzyme inhibitors). It induces the adhesion to the intestinal mucosa and enables a longer retention time at the specific site of drug release [236];
- a backing layer with water impermeable polymers that prevents drug release in the lumen and thus protects it from enzymatic degradation [237,238].

A two-layer patch does not have the pH-sensitive layer, whereas a four-layer patch generally has separated mucoadhesive and drug reservoir layers. Patches can have different sizes in order to better target the surface area for attachment (millimeters or micrometers), they are non-toxic to intestinal tissues, and due to their strong adhesion, they can resist shear forces [237].

Intestinal patches have already been developed to release various biotherapeutics such as insulin [21,239], exenatide [20], salmon calcitonin [240], interferon-α [241], erythropoietin [242], and human granulocyte-colony stimulating factor (G-CSF) [243]. Mucoadhesive devices composed of a mix of FDA-approved polymers (carbopol, pectin, and sodium carboxymethylcellulose) have, for example, triggered a controlled release of insulin and exenatide, with an increased absorption of 13- and 80-fold, respectively, compared to intestinal injections [20]. Insulin-loaded intestinal patches of 700 µm inserted into enteric capsules have also exerted a rapid drug release in 30 min, as well as a significant decrease in blood glucose levels in vivo in rats. Moreover, the addition of other adjuvants (e.g., PEs, protease inhibitors) to the intestinal patch design increased their efficacy [21]. In conclusion, these mucoadhesive delivery devices are promising and have demonstrated their potential in vitro and in vivo. However, further evaluation of their safety should be performed before considering their chronic use in humans.

#### 4.3.3. Intestinal Hydrogels

**Figure 12:**
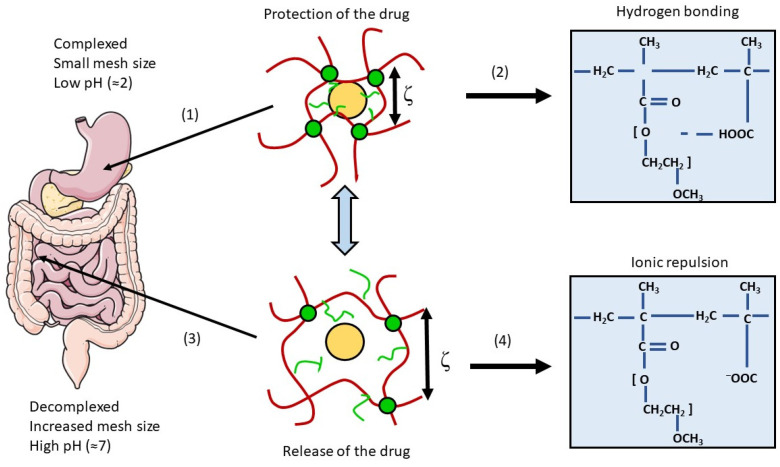
Principle of a stimuli-responsive hydrogel targeting the small intestine. (1) Protection of the drug from the low pH and the proteolytic enzymes of the stomach thanks to (2) a complexation due to hydrogen bonding between the polymer chains; (3) drug release of the drug molecule in the small intestine thanks to (4) decomplexation and an increase in mesh size induced by ionic repulsion and swelling at a higher pH. Figure was adapted from [244]. The left part was modified from Servier Medical Art, licensed under a Creative Common Attribution 3.0 Generic License. http://smart.servier.com/ (accessed on 16 March 2023).

Hydrogels have a 3D network of hydrophilic cross-linked polymers and a water-swollen ability, making them absorb and retain water and biologic fluids in specific environmental conditions without dissolving themselves [244]. Indeed, they are able to respond to variations in the environment to promote a site-specific release of the drug and protect it from the harsh conditions of the GI tract. These abilities are due to a decomplexation (increase in mesh size triggered by ionic repulsion) and a swelling occurring at a specific pH [245,246] (Figure 12).

Hydrogels can be composed of synthetic or natural polymers, as well as a combination of both. They can have different sizes, architectures, and they have a high biocompatibility for biological applications, as their ability for water absorption gives them physical similarities with living tissues (e.g., soft consistency, low interfacial tension) [247].

The pH-responsive hydrogels can be either anionic or cationic. Contrary to cationic hydrogels developed for stomach delivery, anionic hydrogels are ideal for small intestine or colon drug delivery. They remain in a collapsed and low-volume state in an acidic pH, and only absorb water and swell for controlled release of the drug after ionization at a higher pH [248,249].

Many hydrogels have been developed as a strategy to improve the oral administration of drugs by exploiting the physiological differences between the regions of the GI tract. For instance, these promising devices have been used to enable a site-specific delivery of insulin. Indeed, hydrogels composed of poly(methacrylic acid-g-ethylene glycol) were able to incorporate the recombinant biological molecule and protect it in vitro from a release in acidic conditions, whereas a rapid release occurred at higher pH (pH = 7.4). Results in fasted rats have then demonstrated the absorption of insulin in the upper small intestine and the associated hypoglycemic effects [250]. Poly(methacrylic acid-g-ethylene glycol) hydrogels have also significantly improved the absorption of calcitonin and interferon β [251].

To overcome the drug-releasing time that can take tens of minutes, superporous hydrogels, which swell very fast in contact with water thanks to interconnected microscopic pores, have been studied [252]. Hydrogels with a scaffold that can be degraded by enzymes have also been considered as an interesting approach [253].

### 4.4. Strategies to Cross the Intestinal Epithelium

#### 4.4.1. Polymeric Particles

Polymeric particles such as NPs (ranging from 1 nm to 1 μm) and microparticles (ranging from 1 μm to 1 mm), already mentioned in previous sections, are colloidal systems composed of solid polymers that can be either natural or synthetic [22]. They are used for several of their features to enhance the oral delivery of biologics. Due to their submicron size, NPs generally show a higher uptake than microparticles and are often preferred to improve the permeability of drugs [254].

Biologics can be adsorbed at the surface of polymeric particles or encapsulated [255]. Hence, to improve the permeability of a drug, two strategies can be used: either the NPs have a ligand at their surface targeting a specific cell surface receptor, or the physicochemical properties of the NPs are modulated to trigger a non-specific uptake/transport. For instance, only NPs smaller than 100 nm are internalized by absorptive cells [256], as it has been demonstrated in in vitro [257] and in vivo [258] studies. Moreover, other surface characteristics, such as NPs’ zeta potential, hydrophobicity, or mucoadhesive properties, can impact their permeability. These physicochemical properties are modulated by the polymers used and the technique of polymeric particle preparation (e.g., solvent evaporation, emulsion polymerization, interfacial polymerization [259]).

Nevertheless, the main strategy used to cross the intestinal barrier remains the conjugation of NPs with a ligand promoting attachment at the cell surface, in addition to optimal physicochemical properties. Thus, receptors involved in the RMT of their natural ligand have been targeted by NPs [260]. Some of these receptors are described in the next section.

#### 4.4.2. Targeting a Specific Cell-Surface Receptor

Since biologics are restricted to the transcellular pathway and that RMT mechanism is not limited by the size of the cargo, the active targeting of a receptor involved in the transcytosis of its natural ligand was considered as a suitable approach by scientists to improve the oral delivery of biotherapeutics. To that end, either the ligands that will trigger the RMT once bound to their receptors are directly conjugated to the biologics, or they are adsorbed at the surface of polymeric particles encapsulating the drugs. Some of the receptors already targeted are presented in this section.

##### Vitamin B12/IF/Cubilin Receptor

Vitamin B12 (or cobalamin) is an essential nutrient cofactor for animals and humans that has been investigated with its receptor to trigger the transport of macromolecules. This vitamin binds the intrinsic factor in the duodenum, a 60 kDa glycoprotein produced in the gastric epithelium and composed of an α and a β domain. Once the complex is formed, the vitamin B12 is inserted between the two domains of the intrinsic factor to form a combined epitope recognized by the cubilin receptor at the apical surface of enterocytes [261]. This binding triggers the internalization of the complex and the transcytosis of the vitamin. Thus, to induce the RMT of an exogenous molecule, several groups have generated conjugates with the vitamin, while being careful to target a region that does not impact the formation of the complex with the intrinsic factor or the targeting of the combined epitope by the receptor.

The group of Russel-Jones has extensively worked on a drug delivery system based on vitamin B12 [262]. For instance, they have demonstrated that stable conjugates with erythropoietin or G-CSF were actively transported across Caco-2 cell monolayers and into the systemic circulation of rats with unchanged bioactivities [15]. In order to increase the amount of transcytosed complexes, Kishore et al. [263] have even associated two strategies: the active targeting of RMT and the use of polymeric particles by conjugating the vitamin B12 with NPs of dextran containing insulin as the protein of interest. The NPs have induced an important anti-diabetic effect in vivo, with a 70% decrease in blood glucose. Therefore, this study proved once again the ability of vitamin B12 to play the role of a shuttle to enhance the transport of biologics. A polymer-based delivery system called CoboralTM (Access pharmaceuticals Inc., Dallas, TX, USA) has been developed following these studies [264].

##### Transferrin/Transferrin Receptor

The strategy used to trigger a RMT mechanism by targeting a specific receptor has not only been considered for delivering recombinant biological molecules across the intestinal epithelium, but also across the blood–brain barrier (BBB). Indeed, although biotherapeutics represent an emerging class of medicine with substantial promise to treat neurological disorders, similarly to the intestinal mucosa, the BBB is an obstacle limiting brain uptake and thus the therapeutic potential of these recombinant biological molecules. The transferrin/transferrin receptor (Tf/TfR) complex has been extensively studied to overcome the BBB and to provide biologics an access to the brain via RMT [265,266,267,268]. Given these promising studies, this complex has also been explored as a potential biological system for the oral delivery of macromolecules.

Tf is a large monomeric glycoprotein of 80 kDa, internalized in several cell types by RME to induce iron uptake [269]. Although it still remains unclear whether TfR, predominantly expressed on the basolateral side of the IECs, enables the RMT of Tf into systemic circulation [270], several studies have demonstrated an improvement in the intestinal transport of macromolecules by using the Tf/TfR complex. For example, insulin conjugated to iron-loaded Tf has been transported across the Caco-2 monolayer, and after oral administration in fasted streptozotocin-induced diabetic rats, the conjugates induced a slow but prolonged hypoglycemic effect in a dose-dependent manner. Moreover, they were still detected in the plasma 4 h after administration [271]. Tf has also been used as a carrier for human growth hormone [16], G-CSF [17], pro-insulin [272] and 100 nm model polystyrene NPs [273].

##### Immunoglobulin G/Nenonatal Fc Receptor

Different Fc receptors are expressed in the body. However, the one inducing the RMT of IgGs across the epithelium is the FcRn, which belongs to the major histocompatibility complex family [274]. Although its presence is limited to the suckling period in rodents, FcRn expression has been extensively demonstrated in adult human intestines [275,276]. IgG/FcRn complexes were thus studied as an option to develop a system enhancing the oral delivery of macromolecules. The vectorial transport of IgGs is described not only as bidirectional [277], but also as pH-dependent, because of their high affinity for the receptor in an acidic environment, which is lost at neutral pH [278,279].

The IgG transport mechanism has been used to enhance the intestinal permeability of the follicle-stimulating hormone (FSH), a non-covalently linked dimeric protein composed of two subunits. Different constructs have been generated with the latter, using the Fc domain of IgG1, either in a single chain or a heterodimer format. The several conjugates were all found in plasma and had extended half-lives compared to FSH alone after oral dosing in neonatal rats [280,281]. In addition, ovarian and testis weight gains have indicated that the constructs were significantly more active than FSH alone. Hence, these data have highlighted not only the possibility of macromolecule transport induced by their fusion to IgGs, but also the capacity of IgGs to increase the serum half-life of a cargo by their binding to FcRn, which prevents rapid excretion in urine via its resorptive action in the kidney [282].

Some investigations have also demonstrated the ability of NP–Fc constructs to be transported in vitro and in vivo across the intestinal barrier. Indeed, the attachment of the Fc domain to NPs loaded with insulin increased their mean absorption efficiency compared to non-targeted NPs. It also induced a prolonged hypoglycemic response in wild-type mice, similar to the one obtained with a subcutaneous delivery of insulin at the same dose [283]. These effects were prevented in mice lacking the receptor, which proved that the transport of NPs was triggered by the FcRn.

#### 4.4.3. Permeation Enhancers

Another strategy imagined to increase the permeability of biologics is the use of PEs. Over 250 different PEs with several modes of action have been tested in intestinal delivery models [284]. Globally, they are used to improve the permeation of molecules either paracellularly by opening the TJs, or transcellularly by increasing epithelial membrane fluidity.

##### Chelating Agents

Chelating agents are often used for their ability to increase paracellular permeability by sequestering calcium ions (Ca^2+^) needed by the TJs, and for their protease inhibitory activities induced by the withdrawal of essential metal ions out of the enzyme structure.

Diethylenetriaminepentaacetic acid (DTPA) conjugated with poly(g-glutamic acid) was, for instance, successfully used to increase the oral delivery of insulin encapsulated in functional pH-responsive NPs [285]. The inhibitory protease activity of the system was validated in proximal intestinal segments by comparing the degradation of the insulin alone (90% within 2 h) to the conjugates, which were substantially protected. It has also promoted insulin absorption throughout the entire small intestine, with a bioavailability around 20%, and induced a prolonged reduction of blood glucose levels [286]. Another successful study was carried out with EDTA, which has a greater affinity for the Ca^2+^ ions necessary for TJ formation than other chelating agents [77]. It was used in co-administration with protease inhibitors in a phase II clinical trial for a protein oral delivery (POD™) technology [287]. Indeed, it is a common approach to associate chelating agents with other enhancers or enzyme inhibitors to avoid the use of high concentrations that could induce cytotoxic effects [288].

In vitro and ex vivo experiments have shown that the chelating activity of citric acid is inefficient in acidic conditions (pH 3–4) [289], or that chitosan can only be used to open TJs in its protonated form [290,291]. Hence, the use of chelating agents can be a promising strategy to enhance intestinal permeability by opening TJs or inhibiting some of the proteolytic enzymes in the GI tract, but the conditions under which they are effective are important to consider. In addition, it is important to remember that this strategy is a non-selective technique regarding the molecules transported through the intestinal barrier.

##### Surfactants

Surface-active agents, or surfactants [292], are widely used to improve the oral permeability of molecules. Their PE role is to destabilize and solubilize the intestinal barrier via their insertion into cell membranes of enterocytes [293]. The disruption leads to a loss of barrier integrity and a subsequent increase in permeability.

Fatty acids, such as sodium caprylate/caprate and its derivatives, are promising PEs as they have a food additive status. They have been used in many clinical trials over the past 30 years. For example, sodium caprylate has been used for the oral administration of octreotide, a somatostatin analogue for acromegaly treatment [294].In addition, a derivative of sodium caprylate, called SNAC (alcaprozate sodium) [23], has been used in the Eligen^®^ technology (Emisphere, Roseland, NJ, USA) employed in the formulation of the oral delivery treatment of semaglutide for type 2 diabetes mellitus [29]. The Eligen^®^ technology uses multiple surfactants to allow a passive transport mechanism by increasing the lipophilicity of the cargo. The cargo drug is believed to be chaperoned across the epithelium via the transcellular route, but the precise process remains unclear. This technology has also been used to enhance the permeation of insulin [293], salmon calcitonin [295], and heparin [296] in preclinical studies, as well as of vitamin B12. That latter study reached phase III of a clinical trial [297].

Numerous other surfactants are used in oral delivery studies. For instance, the anionic sodium lauryl sulfate has been used to induce an important enhancement of human calcitonin [298] and insulin [299] absorption by disorganizing the membrane architecture and lipid structure. Bile salts are also widely studied because of their biocompatibility. These amphiphilic molecules with a steroid skeleton deriving from cholesterol are capable of increasing the fluidity of cell membranes [300,301]. Several bile salts such as sodium deoxycholate [302], sodium glycodeoxycholate [303], or sodium taurodeoxycholate have thus been studied to improve drug permeability. For example, the latter bile salt cited has been employed in proliposomes to enhance salmon calcitonin absorption. The proliposomes with the bile salt administered intra-duodenally to rats induced a 7.1-fold increase in the bioavailability of salmon calcitonin [304]. Bile salts have also been used for the oral delivery of insulin [305,306,307].

##### Cell-Penetrating Peptides

CPPs are a promising approach to overcome the intestinal membrane barrier as they are able to penetrate into various cell types, via an endocytotic or a non-endocytotic pathway [308], and improve the transport of a large range of molecules [309,310]. Numerous CPPs, with different chemical structures and conformations, have thus been explored due to their penetration ability and their low toxicity at effective concentrations [311,312]. CPPs can be either natural fragments of peptides and proteins, completely synthetized, or chimaeras. They can also be divided into polycationic peptides (such as HIV-1 Tat [313] and oligoarginine peptides [314], both rich in arginines and/or lysines [315]) or amphipathic peptides such as penetratin. CPPs rich in arginines and lysines, which are positively charged, facilitate electrostatic interactions with negatively charged cell surface molecules. CPPs can also contain hydrophobic domains (from amino acids such as tryptophan) allowing their membrane translocation through the lipid bilayer [77].

Several encouraging studies have been carried out to promote the oral administration of biologics after the promising results obtained with Tat as a delivery system for β-galactosidase to several tissues [316]. For example, insulin has been linked to the Tat peptide, which increased the absorption efficiency of the intact macromolecule in Caco-2 cells six to eight times [317]. The same effect was observed with the co-administration of insulin with penetratin [318,319], which increased intestinal bioavailability to 35% (327), and with its analogue PenetraMax^®^ [24]. These studies have demonstrated for the first time the enhancement of insulin absorption based on a non-covalent intermolecular interaction system. However, even if CPPs are an interesting emerging technology to improve membrane permeability, they still need to be validated in clinical studies and they can be sensitive to the difficult proteolytic conditions of the GI tract [320,321].

In summary, a wide range of PEs has been developed and studied over the years, to enhance the oral permeability of recombinant biological molecules. Some of them are even used within the formulation of marketed products. Nevertheless, absorption enhancers cannot usually be given at high concentrations due to their potential toxicity [284]. Hence, to consider the safety and regulation of PEs, they are often combined with other strategies.

#### 4.4.4. Devices for Physical Delivery

##### Microneedles

Microneedle-based technology, an approach extensively used for the transdermal drug delivery of pharmaceutical or cosmetic products [322,323], has been considered for oral drug delivery as it would enable a direct insertion of the biotherapeutics into the intestinal mucosa. Indeed, compared to the skin, the GI tract has a rather insensate nature, allowing painless microinjection. It also has the ability to tolerate the passage of sharp objects and mucosal or epithelial disruptions [324]. In addition, the GI tract can be easily repaired thanks to the high turnover of mucus and IECs. Therefore, these features represent an opportunity for the development of intestinal microneedles, which won’t induce pain during microinjections directly into the intestinal epithelium.

A study realized in 2014 established a proof of concept in swine, demonstrating that microneedle technology could be a safe and efficient strategy for the oral administration of macromolecules such as biologically active insulin. Indeed, a device of 2 cm in length and 1 cm in diameter with 25G needles, and a metallic core for a rapid radiographic detection, was used for microinjections in several parts of the GI tract. The blood-glucose response kinetic of the insulin with this approach was significantly higher than subcutaneous delivery and no evidence of tissue damage was noted [325]. Thus, this study has highlighted the promising future of microneedles for the oral delivery of biologics.

**Figure 13:**
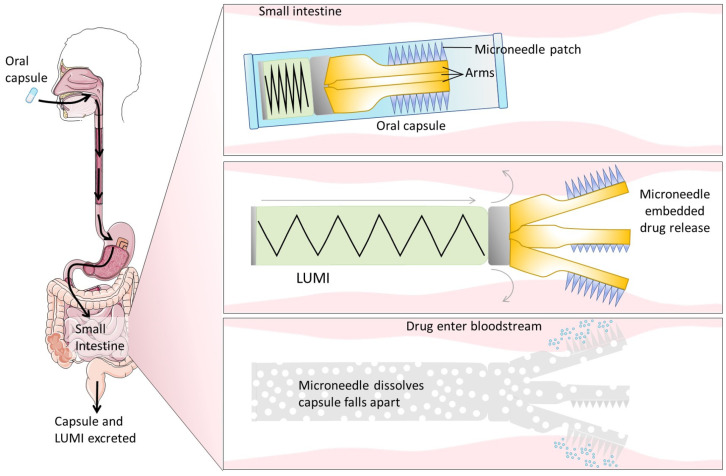
Schematic representation of the luminal unfolding microneedle injector (LUMI). Once the capsule is ingested, the LUMI is ejected in the small intestine and the arms press the patches on the gut wall. The microneedles dissolve and release the encapsulated drug. Then, the capsule breaks apart, and the device rapidly biodegrades before being eliminated from the body. Figure was adapted from [327]. The left part was modified from Servier Medical Art, licensed under a Creative Common Attribution 3.0 Generic License. http://smart.servier.com/ (accessed on 27 December 2022).

Furthermore, metal can be avoided with the use of biodegradable and dissolvable microneedles to increase the biocompatibility of the devices [326]. For example, Abramson et al. have recently developed an oral delivery device combining the technologies of microneedles and intestinal patches, which physically inserts drug-loaded microneedles made of water-soluble materials into the small intestine. This device is called luminal unfolding microneedle injector (LUMI) (Figure 13). It is inserted into ingestible capsules (9 mm in diameter, 30 mm in length) coated with poly(methacrylic acid-co-ethyl acrylate), which dissolve at a pH greater than 5.5. The capsules also use 3.5 kDa PEG as a coating agent to encapsulate the compressed spring, propelling the LUMI out of the capsules in the small intestine. Once the injector is pushed out of the capsule, three degradable arms, which all have microneedle patches of 1 mm length at their far end, unfold outward and press the patches against the intestinal wall. Then, loaded microneedles penetrate the epithelium and dissolve to enable the release of the drug. Promising results were obtained with LUMI loaded with insulin (as a model molecule) which provided a faster pharmacokinetic uptake profile and a systemic uptake superior to 10% of that of a subcutaneous injection over a 4 h sampling period. It also induced an important decrease in blood glucose [25,327]. In addition, the device was able to deliver the microneedles to the intestinal tissues without complete thickness perforations.

An American company (Rani Therapeutics, San Jose, CA, USA) has developed a related technology based on ingestible pills containing a robotic auto-injector. With this technique, the dissolution of the capsule coated with cellulose in the small intestine triggers the inflation of a balloon attached to dissolvable hollow drug-loaded needles. This inflation leads to painless injections into the intestinal wall. Clinical trial studies have demonstrated not only that these robotic pills are safe and well tolerated, but also that they enabled the delivery of a surprisingly high amount of a model molecule (octreotide), with a 65% bioavailability in humans [328]. Therefore, the development of microneedles for the GI tract is an emerging approach with a clinical impact. However, even if this technology is promising, further clinical investigations need to be carried out to ensure the safety and reliability of these devices for long-term administration.

##### Ultrasounds

Ultrasounds are able to manipulate the mechanical energy and transport it as acoustic waves. Hence, they have been extensively studied to overcome the stratum corneum barrier and enable transdermal drug delivery. They were considered as a valuable strategy for their ability to reversibly permeabilize tissues through a phenomenon known as transient cavitation, as well as for their low cost, their non-ionizing and non-invasive characteristics, and other properties [329]. Initially, the investigations focused on high-frequency ultrasounds (≥1 MHz) (470), while the low-frequency ones (20–100 kHz) were only studied in the late 1980s [330,331]. Low-frequency ultrasounds have improved the administration of recombinant biological molecules such as insulin, interferon γ, and erythropoietin across human skin [332]. That is why, heir study has been recently extended to the delivery of biotherapeutics across the small intestine.

Schoellhammer et al. have focused their investigations on the need to develop a rapid, effective, and direct drug delivery system in diseased tissues to treat GI pathologies. Thus, they have evaluated the delivery of model molecules of different molecular weights by using short 1 min-ultrasound treatments ex vivo and in vivo in the GI tract of rodents and pigs [333]. They demonstrated that 1 min of ultrasound exposure enhanced not only the delivery of small molecules (glucose, inulin, hydrocortisone, and mesalamine), but also the delivery of both 3 and 70 kDa dextran. The same research group reported that rectal ultrasounds associated with medicated enema with mesalamine reduced the severity and duration of colitis in rodents more than the usual treatment (free drug enema) and the administration of mesalamine alone. They also proved that 1 min of rectal ultrasound treatments with an insulin enema (macromolecule model) decreased blood glucose in pigs by inducing transient cavitation, whereas no effects were visualized in the absence of ultrasounds. Both in mice and pigs, ultrasounds were well tolerated and safe, as they induced only minor tissue disruptions and did not affect histology, fecal score, or tissue inflammatory cytokine levels.

Therefore, ultrasounds appear to be a safe and efficient technology. Nevertheless, further studies are required to validate their use for a long-term treatment. Furthermore, this device is limited to diseases that require delivery through the rectum due to its current format. Thus, the miniaturization of this technology is a prerequisite to lead to the development of ingestible ultrasound-emitting capsules for systemic delivery [333]. Miniaturization has been recently investigated by Stewart et al., who realized a proof-of-concept study with a capsule device applying focused ultrasounds in the GI tract for the delivery of molecules [334]. The device has been tested ex vivo and in vivo with fluorescent markers (quantum dots). However, in vivo results in swine have demonstrated that the particles were able to penetrate the mucus layer but not to cross the mucosal barrier because of their retention. Hence, although this study has pushed forward the miniaturization of ultrasounds, further optimizations are required to obtain an oral delivery system for biotherapeutics.

To conclude, numerous strategies have been imagined to increase not only the stability of biologics, but also their permeability across the intestinal epithelium. Some of them are already used in marketed products, whereas others are in clinical trials, or further optimized. Table 3 presents a summary of the main goals and limitations of the different approaches described.

## 5. Current Landscape of Clinical Trials and Marketed Orally Delivered Biologics

Despite the tremendous potential of biotherapeutics, the development of a drug candidate suitable for human testing from preclinical studies is arduous, expensive, and time-consuming. First, the optimal dose of delivered biologics to obtain a therapeutic effect is typically difficult to determine and more important than for small molecule drugs because of the low bioavailability impacting clinical efficacy [30,337]. Moreover, due to their complex nature, biologics are more vulnerable to environmental factors [338,339]. They also require specialized and costly production processes involving living cells and purification steps that are inherently more variable than components involved in chemical synthesis. Manufacturing hurdles are also dependent on the final form chosen. For instance, for a liquid product at high concentration, viscosity is the main issue to overcome since it may impact the quality of the drugs by disturbing some processes (e.g., filtration), whereas for solid drugs, the lyophilization step introduces a risk of degradation (e.g., through protein aggregation) [340]. Hence, clinical translation is paved with obstacles, and bringing biologics from the bench to the marketplace is challenging and expensive. As a result, and despite the intensive research, only a few molecules are commercially available.

One of the few molecules approved by the FDA is the cyclosporin A, commercially available under the names Neoral^®^/Sandimmune^®^, and developed by Novartis (Switzerland) as immunosuppressants. Neoral^®^ is a newer formulation of cyclosporine that uses microemulsion technology with a mixture of oils and surfactants to enhance the bioavailability of the biological drug. With this strategy, the absorption of the drug is more consistent and predictable, which helps in decreasing the variability in cyclosporine blood levels that were prevalent in older versions such as Sandimmune^®^ [341,342]. More recently, in 2019, oral semaglutide gained approval by the FDA and is marketed as Rybelsus^®^ by Novo Nordisk (Plainsboro, NJ, USA). It is a glucagon-like peptide-1 receptor agonist used for the treatment of type 2 diabetes mellitus, by stimulating the secretion of insulin and suppressing glucagon secretion, resulting in improved glycemic control. It uses the Eligen^®^ technology mentioned in the previous section for the formulation. Another example of a marketed biologic for oral delivery is the octreotide from Chiasma (Needham, MA, USA), marketed under the name Mycapassa^®^. This medication was approved by the FDA in 2020 for the long-term maintenance treatment of acromegaly in patients who have responded to and tolerated treatment with octreotide or lanreotide [294]. The used Transient Permeation Enhancer^®^ (TPE^®^) technology consists of an oily suspension of medium-chain fatty acid salts inserted in soluble hydrophilic microparticles coated with an enteric coating that dissolves within intestinal fluids [28]. Desmopressin acetate (DDAVP^®^, Ferring Pharmaceuticals, St-Prex, Switzerland), used for diabetes insipidus, and Taltirelin hydrate (Ceredist^®^/Ceredist OD^®^, Mitsubishi Tanabe Pharma Co., Osaka, Japan), for spinocerebellar degeneration, are two other orally commercially available biologics [343,344,345,346].

Even if there are very limited oral biotherapeutics on the market, multiple molecules formulated with different technologies are currently being clinically evaluated for systemic delivery. Among them, oral insulin is extensively studied. A non-exhaustive list of orally administered biotherapeutics that have been recently tested are listed in Table 4. These trials represent the future prospects of the oral delivery of biologics. Nevertheless, as it has been demonstrated by the oral insulin of Oramed Pharmaceuticals Inc. (Jerusalem, Israel) [27], which, despite great hopes, failed to beat placebo in a phase III trial, the road to success remains challenging [347].

## 6. Conclusions

The stability and permeability of orally delivered biologics are impeded by the physiological barriers of the GI tract. Indeed, once ingested, biologics first have to overcome the harsh conditions of the stomach and the numerous secreted proteases and peptidases of the small intestine. Then, their absorption is hindered by mucus entrapping the macromolecules, and by the underlying intestinal epithelium, whose TJs restrict the paracellular transport. The size and hydrophilicity of recombinant biological molecules also limit their passive transcellular diffusion through epithelial cells.

To support the development and optimization of orally delivered biotherapeutics, in vitro models have been developed. Cell-based techniques are usually simple, practical, and enable the rapid assessment of the transport of molecules. Nevertheless, they do not always closely mimic human physiology. Thus, to overcome some of the intrinsic drawbacks of monocultures, co-cultures and 3D culture models have been studied. However, despite considerable efforts devoted to the development of in vitro techniques, it is still difficult to reproduce the complexity and all the features of the intestinal mucosa. Consequently, to accurately predict drug permeability, a combination of in vitro and ex vivo approaches is recommended. Indeed, tissue-based models are not suitable for preliminary screenings because of their low throughput and their more complex set-ups. Nevertheless, they represent an interesting compromise between in vitro and in vivo experimental models by allowing intestinal transport studies on viable tissues.

Thanks to the different in vitro and ex vivo models developed, progress has been made in the understanding of the transport mechanisms and physiological barriers impacting the bioavailability of biologics. These discoveries led to the development of various strategies, allowing researchers to overcome obstacles impacting the integrity of molecules or hindering their transport. To increase the stability and the absorption across the intestinal mucosa, while remaining well tolerated and safe in vivo, several of these innovative techniques are often combined. That is indeed the case for most of the clinically tested or marketed biological products orally delivered, such as MYCAPSSA^®^ associating an enteric coating, surfactants, and polymeric particles; the POD™ technology using an enteric coating, protease inhibitors, and chelating agents; or the technology developed by Rani Therapeutics using microneedles inserted into robotics pills with an enteric coating.

Nevertheless, despite all these discoveries and improvements, the number of FDA-approved orally administered biologics remains low, which emphasizes how oral delivery is a challenging topic that still requires more attention and further investigations.

## Figures and Tables

**Table 3:** Main goals and limitations of strategies developed to improve the oral delivery of biologics.

Parameter to Improve	Strategy	Goals	Limitations	References
Stability	Enteric coating	To bypass the harsh gastric conditions	Not sufficient alone to protect from the biochemical barrier of the GI tract	[7,77,198]
	pH modulators	To modulate the microenvironment’s pH in order to hinder the activation of proteolytic enzymes	Can influence the dissolution of enteric coating, shifts in pH could alter the cargo	[7,77,203,204]
	Enzyme inhibitors	To reduce the activity or inactivate proteolytic enzymes by the binding to their specific site	Possible side effects in long-term therapies (e.g., absorption of unwanted molecules, damages to the GI tract)	[205,211,212]
Mucus penetration	Mucus penetrating systems	To cross the mucus layer in order to reach epithelial cells (mucolytics, NPs)	Not sufficient alone to increase intestinal permeability, side effects for mucolytics	[34,219,224]
Contact time/site-specific delivery	Thiomers	To increase contact time with the mucosa by binding to the mucus	Prone to oxidation, dependent on the mucus turn-over	[77,226,231]
	Intestinal patches	To create a high concentration gradient, to increase the residence time at the site-specific drug release, and to confer protection from the harsh GI environment	Importance of the formulation (e.g., size, polymers) to avoid side effects	[233,234,237]
	Hydrogels	To promote a site-specific release and to confer protection from harsh GI environment	Swelling dependent on diffusion of water	[244,245,248]
Permeability	NPs	Among others, to cross the intestinal epithelium by modulating optimal physicochemical properties and/or active targeting of a receptor	Depending on the polymers used: toxicity or instability of the drugs	[22,256,335,336]
	Targeting of receptors	To trigger RMT across the intestinal epithelium	Optimal properties of the ligands to trigger RMT unknown (e.g., affinity, epitope targeted)	[7,262,270,271,280]
	PEs	To increase paracellular or transcellular transport across the intestinal barrier	Toxicity at high level concentrations, non-selective techniques	[284,285,308]
	Microneedles	To physically deliver the molecules via insertion into the intestinal mucosa	Potential use of metal, safety in long-term therapies not validated, amount of drug that can be loaded per microneedle	[324,325,328]
	Ultrasounds	To permeabilize tissues (reversibly)	Miniaturization of the device that needs further optimization	[329,333,334]

**Table 4:** Examples of orally delivered biologics in clinical trials (clinical trials.gov).

Biological Molecule	Company	Indication	Strategies
Insulin (Phase III: NCT04754334)	Oramed Pharmaceuticals (Jerusalem, Israel)	Type 2 Diabetes Mellitus	PE in insulin prodrug
Insulin (Phase II/III: NCT00814294 and NCT03096392)	Diasome Pharmaceuticals Inc. (Cleveland, OH, USA)	Type 2 Diabetes Mellitus	Liver-targeted liposomes
Insulin (Phase II/III: NCT03430856)	Biocon Ltd. (Bangalore, Karnataka, India)	Type 1 Diabetes Mellitus	PE (sodium caprate) included as permeability enhancer in insulin prodrug
Insulin (Phase II: NCT01973920)	Oshadi Drug Administration Ltd. (Rehovot, Israel)	Type 1 Diabetes Mellitus	NPs
Insulin (Phase II: NCT02470039)	Generex Biotechnology Corp. (Burlington, Canada)	Type 2 Diabetes Mellitus	Microemulsion systems (fatty acids) and enteric coating
Parathyroid hormone (PTH) (Phase II: NCT03516773)	Proxima Concepts Ltd./Diabetology (London, UK)	Hypoparathyroidism	PEs
Salmon calcitonin (Phase III: NCT00959764)	Tarsa Therapeutics (Philadelphia, PA, USA)	Osteoporosis, Postmenopausal	pH modulator
Leuprolide (Phase II: NCT05096065)	Enteris Biopharm (Boonton, NJ, USA)	Endometriosis	PE, pH modulator, and enzyme inhibitor

## Data Availability

Data sharing not applicable.
